# Evaluation of use of the optional unit QA-810V for the determination of five-part leukocyte differentials

**DOI:** 10.1155/S1463924601000098

**Published:** 2001

**Authors:** Izumi Tsuda, Takayuki Takubo, Tomio Kamitani, Noriyuki Tatsumi

**Affiliations:** 1 Department of Clinical and Laboratory Medicine Osaka City University Medical School 1-4-3 Asahimachi Abeno Osaka 545-8585 Japan; 2 Wakakoukai Hospital Japan

## Abstract

The newly developed QA-810V is an optional unit for the determination of five-part white blood cell differentials. It can be used together with the same manufacturer's haematology analyser which has been used in relatively small-sized laboratories. The present study evaluates the basic performance of the QA-810V and the MEK-8118 haematology analyser using routinely obtained blood specimens treated with ethylenedioaminetetraacetic acid-2K. In this evaluation, reproducibility was good and little carryover was found. Accurate measurements were possible for up to 24h of storage. Storage at 4 ^°^C yielded more stable measurements of complete blood counts and five-part differentials than storage at room temperature. A good correlation between findings with the MEK-8118 haematology analyser and those with the SE-9000 haematology analyser was found for complete blood counts. The leukocyte differential obtained with the QA-810V correlated well with eye counts, with r > 0.9 for percentages of neutrophils, lymphocytes and eosinophils. Scattergrams obtained with the QA-810V reflected the presence of abnormal cells. The performance of the QA-810V was excellent and it can improve the quality of testing in clinical laboratories.


Journal of Automated Methods & Management in Chemistry, Vol. 23, No. 3 (May–June 2001) pp. 77–81

### Evaluation of use of the optional unit QA-810V for the determination of five-part leukocyte differentials

**Izumi Tsuda[1*], Takayuki Takubo[1], Tomio Kamitani[2] and Noriyuki Tatsumi[1]**

*[1]Department of Clinical and Laboratory Medicine, Osaka City University Medical School, 1-4-3 Asahimachi, Abeno, Osaka, 545-8585 and [2]Wakakoukai Hospital, Japan*

*The newly developed QA-810V is an optional unit for the determination of five-part white blood cell differentials. It can be used together with the same manufacturer's haematology analyser which has been used in relatively small-sized laboratories. The present study evaluates the basic performance of the QA-810V and the MEK-8118 haematology analyser using routinely obtained blood specimens treated with ethylenedioaminetetraacetic acid-2K. In this evaluation, reproducibility was good and little carryover was found. Accurate measurements were possible for up to 24 h of storage. Storage at 4°C yielded more stable measurements of complete blood counts and five-part differentials than storage at room temperature. A good correlation between findings with the MEK-8118 haematology analyser and those with the SE-9000 haematology analyser was found for complete blood counts. The leukocyte differential obtained with the QA-810V correlated well with eye counts, with r > 0.9 for percentages of neutrophils, lymphocytes and eosinophils. Scattergrams obtained with the QA-810V reflected the presence of abnormal cells. The performance of the QA-810V was excellent and it can improve the quality of testing in clinical laboratories.*

### Introduction

Haematology analysers with five-part leukocyte differential function are now used in sophisticated laboratories. On the other hand, some small laboratories still use haematology analysers without the five-part differential function; the reasons for this may vary, but they include the large investment required for the latest instrumentation, the small number of specimens assayed for the five-part differential, and the limited space in such laboratories. Haematology analysers can screen for abnormal samples that require further manual differential, and the usefulness of automated leukocyte differential for this purpose is recognized [1]. In addition, screening by the haematology analysers with the function of the five-part differential is superior to those with the three-part differential or without automated differential [2, 3]. The recently developed QA-810V (QA, Nihon Kohden, Tokyo, Japan) is an optional unit with a five-part differential function designed to be used with Nihon-Kohden's haematology analyser with the function of three-part differential. The investment required can be minimal for users of these haematology analysers. The cost and size of the optional unit and the optional unit together with haematology analyser are for use in relatively small laboratories. The present study performs a basic evaluation of use of both the QA-810V and MEK-8118 haematology analyser (MEK, Nihon Kohden).

* e-mail: noritatsumi@med.osaka-cu.ac.jp

### Materials and methods

### Materials

Venous blood treated with ethylenedioaminetetraacetic acid-2K was processed within 6 h after blood collection, except for stability tests. The samples were randomly selected from the laboratory routine workload.

### Instruments

Complete blood counts (CBC) are determined with the MEK analyser. Counting of white blood cells, red blood cells and platelets is based on the impedance method. Haemoglobin is determined with the haemoglobin-cyanide method, an internationally recognized standard method. For determination of leukocyte differential, leukocytes were suspended in the diluent in the instrument, and the cells in a flow cell were analysed from three angles using a semiconductor laser light. Cell granularity was analysed with orthogonal scatter, and the complexity in the cell was detected with a forward high-angle scatter. Cell size was determined with a forward low-angle scatter. By combining the above information, the five-part differential was obtained.

The 22 parameters obtained with the MEK and QA analysers were: white blood cell count, red blood cell count, haemoglobin, haematocrit, mean corpuscular volume, mean corpuscular haemoglobin, mean corpuscular haemoglobin concentration, red cell distribution width, platelet count, mean platelet volume, plateletcrit, platelet distribution width, and percentages and absolute counts of the five subpopulations of white blood cells.

The MEK with QA was calibrated before this evaluation and checked with quality control material daily before the experiments.

### Reproducibility

Samples ($n = 10$) were assayed five times, and the coefficients of variation (CoV) were obtained. The mean of the CoVs was calculated for each parameter.

*Carryover test*

Triplicate measurements of a high-count ($a_{1-3}$) sample followed by a low-count ($b_{1-3}$) sample were performed, and the carryover effect was determined using:

$$\text{Carryover} = [b_1 - (b_2 + b_3)/2]/[(a_2 + a_3)/2].$$

*Stability during storage*

Samples from healthy adults ($n = 3$) were stored for up to 48 h, and the changes in the obtained parameters during storage were determined. Each sample was stored in two tubes, one at 4°C and one at room temperatures (25°C), and measured at 0, 4, 8, 12, 24 and 48 h after blood collection. The samples stored at 4°C were left at room temperature for 10 min before measurements. Changes were evaluated as the mean of actual values of three samples at each time assayed and the percent changes in parameters calculated for each sample with the percentage at 0 h set as 100%.

*Comparability with other methods*

The samples were processed on the MEK with QA and on the laboratory's routine haematology analyser (SE-9000, Sysmex Corp., Kobe, Japan). The CBC obtained with the two instruments were compared. For leukocyte differentials, manual differential counts routinely performed (100 counts) were used for comparison. Medical technologists with more than 10 years of experience performed 100 cell differential counts on May–Grünwald–Giemsa-stained peripheral blood films prepared by the wedge method.

### Results

*Reproducibility*

The mean CoVs for CBC varied from 0.5 to 2.5% (table 1). For leukocyte differential percentages, CoVs ranged from 1.9% for neutrophils to 45.2% for basophils.

*Carryover*

The mean high and low white blood cell counts were 19.63 and 0.6 ($\times 10^3/\mu l$); those for red blood cell count were 5.15 and 0.97 ($\times 10^6/\mu l$); and those for platelet count were 558.0 and 21.7 ($\times 10^3/\mu l$). Carryover for white blood cell count was 0%, while those for both red blood cell and platelet counts were 0.68 and 0.36% respectively.

*Stability during storage at 4°C and room temperature* (table 2)

The CBC parameters were stable for up to 24 h of storage regardless of temperature. After 24 h of storage, mean corpuscular volume tended to increase at room temperature but was stable at 4°C. Changes in other parameters were larger, although percent changes were < 4% at 4°C and ~5% at room temperature.

The percent changes up to 48 h were within 10% for %neutrophils and %lymphocytes. The changes in other subpopulations appeared to be high, but the actual changes in percentages in differentials were < 1% for 12 h of storage at both temperatures. Even for storage of 24 h, the actual changes were at most 3.7%.

*Comparability with other methods* (table 3)

The correlations for five major CBC parameters ($n = 768$) between results obtained with the MEK and SE-9000 analysers are shown in table 2. Correlation coefficients ranged from 0.984 to 0.997. Leukocyte differential findings obtained with the QA and the manual method were compared for 237 samples. The correlation coefficients for %neutrophils, %lymphocytes and %eosinophils were > 0.9 for the two methods. For %basophils, $r$ was low, although the range of variation for %basophils was narrow.

*Scattergrams*

Figure 1 (left) shows a scattergram of the combination of size and complexity for a normal sample. Lymphocytes, monocyte and basophils were distributed in the left area,

*Table 1. Reproducibility test.*

| | CoV (%) | | Mean | |
|---|---|---|---|---|
| *Parameter (unit)* | *Mean ± SD* | *Min–Max* | *Mean ± SD* | *Min–Max* |
| WBC ($\times 10^3/\mu l$) | 1.29 ± 0.31 | 0.90–1.83 | 5.09 ± 0.92 | 3.78–6.98 |
| RBC ($\times 10^6/\mu l$) | 0.70 ± 0.34 | 0.24–1.32 | 4.24 ± 0.49 | 3.26–4.85 |
| Hgb (g/dl) | 0.56 ± 0.27 | 0.31–1.21 | 13.17 ± 1.42 | 10.36–14.76 |
| Hct (%) | 0.64 ± 0.29 | 0.31–1.05 | 38.96 ± 3.88 | 30.74–43.54 |
| Plt ($\times 10^3/\mu l$) | 2.48 ± 0.90 | 1.20–4.03 | 186.82 ± 65.43 | 59.20–266.40 |
| Lym% (%) | 2.27 ± 1.08 | 1.13–4.85 | 34.78 ± 7.79 | 26.18–46.34 |
| Mon% (%) | 8.05 ± 3.96 | 3.53–16.32 | 5.90 ± 1.55 | 4.10–9.14 |
| Neu% (%) | 1.90 ± 1.03 | 0.41–3.43 | 54.31 ± 8.47 | 39.90–64.98 |
| Eos% (%) | 8.75 ± 5.54 | 2.76–21.22 | 4.74 ± 2.16 | 1.96–8.74 |
| Bas% (%) | 45.18 ± 24.34 | 20.33–100.00 | 0.26 ± 0.15 | 0.10–0.60 |

SD, standard deviation; CoV, coefficient of variation; WBC, white blood cell count; RBC, red blood cell count; Hgb, heamoglobin; Hct, haematocrit; Plt, platelet count; Lym%, percent lymphocytes; Mon%, percent monoocytes; Neu%, percent neutrophils; Eos%, percent eosinophils; Bas%, percent basophils.

*Table 2. Stability of complete blood count (CBC) and leukocyte differential results.*

| | | Time (h) | | | | | |
|---|---|---|---|---|---|---|---|
| | | 0 | 4 | 8 | 12 | 24 | 48 |
| *CBC*: | | | | | | | |
| RT | WBC | 5.67 | 5.67 | 5.63 | 5.63 | 5.67 | 5.37 |
| | % change | 100.00 | 100.00 | 99.28 | 99.42 | 100.00 | 94.98 |
| | RBC | 4.93 | 4.88 | 4.88 | 4.87 | 4.87 | 4.78 |
| | % change | 100.00 | 99.02 | 98.97 | 98.69 | 98.70 | 96.97 |
| | Hgb | 16.03 | 15.87 | 15.77 | 15.67 | 15.83 | 15.60 |
| | % change | 100.00 | 98.94 | 98.32 | 97.69 | 98.73 | 97.24 |
| | MCV | 94.23 | 94.33 | 94.37 | 94.37 | 96.67 | 101.07 |
| | % change | 100.00 | 100.10 | 100.14 | 100.14 | 102.58 | 107.25 |
| | Plt | 184.00 | 184.33 | 182.33 | 182.67 | 186.33 | 173.33 |
| | % change | 100.00 | 100.08 | 98.98 | 99.24 | 101.34 | 94.45 |
| 4 C | WBC | 5.77 | 5.70 | 5.67 | 5.70 | 5.70 | 5.57 |
| | % change | 100.00 | 99.18 | 98.46 | 98.94 | 98.94 | 96.59 |
| | RBC | 4.93 | 4.90 | 4.93 | 4.91 | 4.93 | 4.87 |
| | % change | 100.00 | 99.28 | 99.84 | 99.56 | 99.98 | 98.67 |
| | Hgb | 16.10 | 16.03 | 15.83 | 15.83 | 16.00 | 15.97 |
| | % change | 100.00 | 99.57 | 98.33 | 98.35 | 99.38 | 99.16 |
| | MCV | 94.80 | 94.43 | 94.50 | 94.40 | 94.50 | 95.23 |
| | % change | 100.00 | 99.61 | 99.68 | 99.57 | 99.68 | 100.45 |
| | Plt | 186.67 | 185.67 | 185.00 | 182.67 | 183.33 | 188.33 |
| | % change | 100.00 | 99.51 | 99.02 | 97.70 | 98.17 | 100.69 |
| *Leukocyte differential*: | | | | | | | |
| RT | Lym% | 34.83 | 35.23 | 35.17 | 34.33 | 33.50 | 49.80 |
| | % change | 100.00 | 101.11 | 100.28 | 97.93 | 95.73 | 162.56 |
| | Mon% | 5.30 | 4.67 | 5.23 | 5.57 | 8.97 | 19.27 |
| | % change | 100.00 | 90.42 | 102.43 | 109.26 | 173.35 | 362.99 |
| | Neu% | 57.43 | 57.50 | 56.70 | 57.73 | 55.33 | 18.50 |
| | % change | 100.00 | 100.12 | 98.63 | 100.55 | 96.95 | 33.43 |
| | Eos% | 2.33 | 2.43 | 2.73 | 2.80 | 5.77 | 10.33 |
| | % change | 100.00 | 125.20 | 149.60 | 137.04 | 336.47 | 619.00 |
| | Bas% | 0.10 | 0.17 | 0.17 | 0.20 | 0.50 | 2.10 |
| | % change | 100.00 | 175.00 | 100.00 | 125.00 | 500.00 | 1500.00 |
| 4 C | Lym% | 32.63 | 32.27 | 32.97 | 31.90 | 30.70 | 30.63 |
| | % change | 100.00 | 98.70 | 101.22 | 97.38 | 93.13 | 93.02 |
| | Mon% | 4.90 | 3.53 | 3.57 | 4.23 | 5.47 | 6.17 |
| | % change | 100.00 | 72.68 | 73.81 | 87.13 | 112.92 | 127.05 |
| | Neu% | 59.73 | 61.20 | 59.77 | 60.10 | 58.77 | 55.33 |
| | % change | 100.00 | 102.49 | 100.11 | 100.64 | 98.48 | 92.60 |
| | Eos% | 2.57 | 2.40 | 2.93 | 2.63 | 2.80 | 3.63 |
| | % change | 100.00 | 93.09 | 126.49 | 100.96 | 113.56 | 152.07 |
| | Bas% | 0.17 | 0.60 | 0.77 | 1.13 | 2.27 | 4.23 |
| | % change | 100.00 | 383.33 | 500.00 | 783.33 | 1466.67 | 2700.00 |

RT, room temperature; WBC, white blood cell count; RBC, red blood cell count; Hgb, haemoglobin; Hct, haematocrit; Plt, platelet count; Lym%, percent lymphocytes; Mon%, percent monoocytes; Neu%, percent neutrophils; Eos%, percent eosinophils; Bas%, percent basophils.

whereas neutrophils and eosinophils were distributed in the right area. Lymphocytes could be differentiated since they were smaller than monocytes and basophils. Monocytes and basophils could be separated by their difference in granularity (figure 1, middle), and neutrophils and eosinophils could be differentiated in the same fashion (figure 1, right). Ghosts were distributed in the lower part of the scattergram. For normal samples, individual clusters of such subpopulations could be clearly observed, but abnormal scattergrams were obtained for samples including abnormal cells. Figure 2A is a scattergram of cord blood with nucleated red blood cells (7/100 white blood cells) and 15% immature granulocytes (myelocytes and metamyelocytes) as determined by manual differential. On this scattergram, the border of the area between lymphocytes and ghosts is difficult to find, since atypical lymphocytes may be distributed there. A large cluster is found covering the area of monocytes and basophils and the area of neutrophils and eosinophils. QA exhibited flagging of 'Left Shift' for this sample. Figure 2B shows a case of malignant lymphoma, for which 1% blasts, 22% immature granulocytes and 17% stab cells were found on manual differential. A large cluster is seen in the upper part of a scattergram. The QA exhibited flagging of 'Left Shift', 'Immature Granulocytes' and 'Blasts' for this sample.

### Discussion

The present study was performed to evaluate the basic function of both the MEK and QA haematology analy-

*Table 3. Comparability of results obtained with the MEK-8118 and QA-810V.*

| | $x$ | $y$ | $n$ | $\bar{x} \pm 1\ SD$ | $\bar{y} \pm 1\ SD$ | $r$ | $y = ax + b$ |
|---|---|---|---|---|---|---|---|
| *Complete blood counts*: | | | | | | | |
| WBC | SE-9000 | MEK | 768 | 6.221 ± 2.977 | 6.326 ± 2.958 | 0.997 | 0.990 $x$ + 0.167 |
| RBC | SE-9000 | MEK | 768 | 4.042 ± 0.673 | 3.995 ± 0.655 | 0.996 | 0.968 $x$ + 0.081 |
| Hgb | SE-9000 | MEK | 768 | 12.296 ± 1.980 | 12.383 ± 1.974 | 0.997 | 0.994 $x$ + 0.159 |
| Hct | SE-9000 | MEK | 768 | 36.953 ± 5.674 | 36.813 ± 5.699 | 0.991 | 0.996 $x$ + 0.022 |
| Plt | SE-9000 | MEK | 768 | 227.217 ± 105.129 | 227.305 ± 102.023 | 0.984 | 0.955 $x$ + 10.278 |
| *Five-part differential of white blood cells*: | | | | | | | |
| Neu % | mannual differential | MEK | 237 | 65.932 ± 18.218 | 64.068 ± 17.614 | 0.931 | 0.900 $x$ + 4.730 |
| Lym% | mannual differential | MEK | 237 | 22.287 ± 15.338 | 24.922 ± 15.827 | 0.940 | 0.970 $x$ + 3.298 |
| Mon% | mannual differential | MEK | 237 | 6.253 ± 4.259 | 6.581 ± 4.159 | 0.740 | 0.723 $x$ + 2.063 |
| Eos% | mannual differential | MEK | 237 | 3.730 ± 6.052 | 3.565 ± 4.580 | 0.932 | 0.705 $x$ + 0.934 |
| Bas% | mannual differential | MEK | 237 | 0.658 ± 0.960 | 0.865 ± 0.789 | 0.132 | 0.109 $x$ + 0.793 |

WBC, white blood cell count; RBC, red blood cell count; Hgb, haemoglobin; Hct, haematocrit; Plt, platelet count; Neu%, percent neutrophils; Lym%, percent lymphocytes; Mon%, percent monocytes; Eos%, percent eosinophils; Bas%, percent basophils.

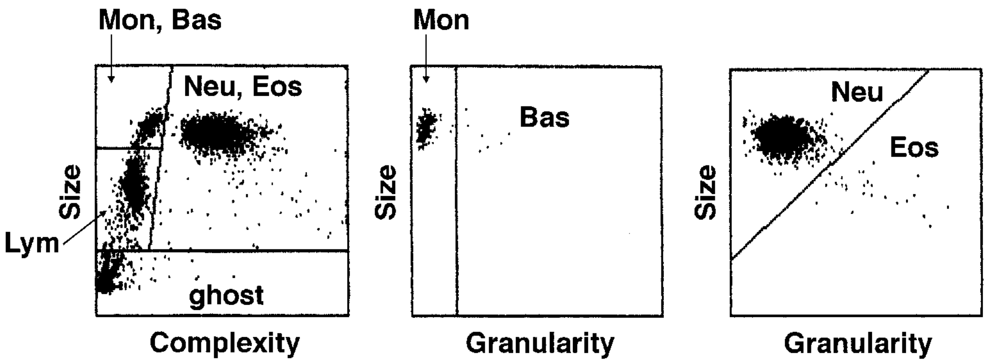

*Figure 1. Normal scattergram. Neu, neutrophils; Lym, lymphocytes; Mon, monocytes; Eos, eosinophils; Bas, basophils.*

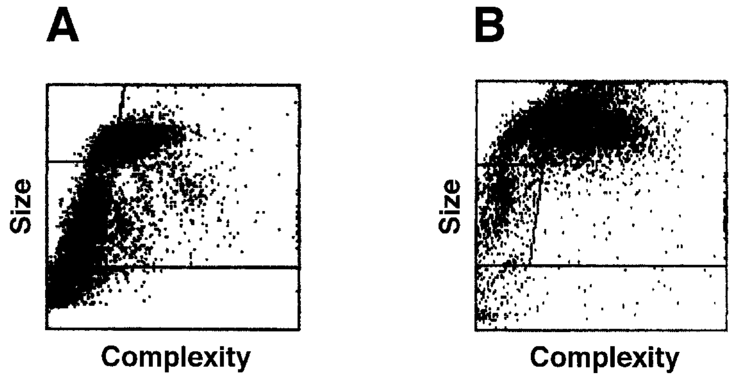

*Figure 2. Abnormal scattergrams. A, cord blood; B, malignant lymphoma.*

sers. Reproducibility and carryover were satisfactory. Stability testing indicated that results were more stable at 4°C than at room temperature for both CBC and leukocyte differential counts and that at 4°C the changes were small even after 24 h of storage. In addition, CBC results obtained with the MEK correlated well with those obtained with the SE-9000, and the five-part differentials obtained with the QA were comparable with manual differential counts, with good agreement between the two in percentages of neutrophils, lymphocyte, monocytes and eosinophils. The coefficient of correlation between manual and automated counts was low for the percentage of basophils, but no significant difference between the two methods was found by a paired $t$-test. Basophils are difficult to quantitate on most haematology analysers because they are relatively few in number. With the

QA, three scattergrams are obtained, and most abnormalities could be detected by observation of the scattergram of complexity and size. This scattergram clearly reveals the position of each subpopulation, and thus should be useful clinically. Overall, the results of the evaluation were favourable and similar to those for other instruments with five-part differentials.

Recent economic constraints have forced clinical laboratories to merge in order to optimize their cost structure. Reimbursement for clinical assays will decrease and clinical laboratories, regardless of their sizes, must correspondingly make significant improvement in efficiency and generate significant cost savings. Thus, clinical laboratories must try to minimize investment, and manufacturers must consider the development of instruments that meet the above users' needs. Manufacturers must develop smaller and more inexpensive instruments [4–6]. At the same time, implementation of quality systems has been emphasized in laboratories. This trend is strong especially in the USA since the National Committee of Clinical Laboratory Standards, the Food and Drug Administration and the College of American Pathologists review the guidelines for haematology testings and plan to revise them to improve quality systems [7, 8]. The effects of these revisions will soon spread to other countries. Haematology systems are expected to meet revised criteria for quality systems, and thus neither instruments that require significant investment nor small but unreliable instruments are any longer required. The QA was for this reason developed for use with the MEK haematology analyser. For users possessing Nihon Kohden's haematology analyser, the investment required for the QA can be minimal (at most one-quarter that required for other haematology analysers with five-part differentials) since users need only purchase the optional unit to improve their quality of screening for morphologically abnormal samples. Even the MEK with QA is still less expensive than other haematology analysers with five-part differentials now on the market in Japan.

In conclusion, the QA will improve clinical laboratory productivity and efficiency, and the increase in the detail of automated examination it enables will improve both the quality and quantity of laboratory testing. A haematology analyser with a maximum capability to generate a reliable automated haematology results, a good understanding of analyser limitations and documented review criteria developed by the laboratory to direct the actions taken based on automated results are essential for the success for good laboratory practice.

### References

1. Van Hove, L., *Laboratory Hematology*, **5**, 1999, 43.
2. Bentley, S. A., *Baillières Clin. Haematol.*, **3**, 1990, 851.
3. Sanzari, M., De Toni, S., D'Osualdo, A., Rossetti, M., Floriani, F. and Plebani, M., *Panminerva Med.*, **40**, 1998, 116.
4. Wood, B. L. and Andrews, J., *Laboratory Hematology*, **5**, 1999, 1.
5. Tsuda, I., Sagawa, H., Takubo, T., Kawai, S. and Tatsumi, N., *Journal of Automatic Chemistry*, **18**, 1996, 163.
6. Tombert-Paolantoni, S., Tourlourat, M., Chretien, M. C., Zaouche, S. and Baufine-Ducrocq, H., *Laboratory Hematology*, **5**, 1999, 59.
7. National Committee for Clinical Laboratory Standards, *A Quality System Model for Health Care*. Approved Guideline NCCLS GP26-A (Villanova: NCCLS, 1999).
8. College of American Pathologists, in A. Rabinovitch (ed.), *Laboratory Accreditation Program Checklist 2: Hematology*, (CAP, 1998).

